# Maximum lifetime body mass index is the appropriate predictor of knee and hip osteoarthritis

**DOI:** 10.1007/s00402-017-2825-5

**Published:** 2017-10-27

**Authors:** Sabine Patricia Singer, Dietmar Dammerer, Martin Krismer, Michael C. Liebensteiner

**Affiliations:** 1 Private Practice for Orthopaedics, Am Isarkanal 24/6/7, 81379 Munich, Germany; 2 Department of Orthopaedic Surgery, Medical University of Innsbruck, Anichstr. 35, 6020 Innsbruck, Austria

**Keywords:** Obesity, Body mass index, Knee osteoarthritis, Hip osteoarthritis, Overweight

## Abstract

**Introduction:**

In light of inconsistencies in the literature, this study aimed to investigate the relationship between obesity (current and historic) and osteoarthritis (OA) of the knee or hip.

**Materials and methods:**

We examined 99 people (knee OA, hip OA and controls), age > 50 years, in a case–control study. The current weight, height and waist circumference were measured on site, and detailed weight changes over their lifetime were based on questionnaires and standardized interviews. We used binomial logistic regression to determine the predictive value for an osteoarthritis group membership of each derived indicator.

**Results:**

An increase in ‘maximum-BMI’ increased the odds ratio for both knee OA (OR 1.2; CI 1.1–1.4; *p* = 0.005; *R* ^2^ = 0.36) and hip OA (OR 1.2; CI 1.0–1.3; *p* = 0.027; *R* ^2^ = 0.16). Current BMI was significantly associated with knee OA but not with hip OA. A high “minimum-BMI” (over the age of 18 years) had the highest odds ratio of all calculated indicators for both osteoarthritis groups.

**Conclusions:**

Based on our findings, it is concluded that the maximum BMI over one’s lifespan is a better predictor of OA of the hip or the knee than the current BMI. The knee joint seems to be more sensitive to obesity as current BMI was associated only with knee OA but not with hip OA.

## Introduction

Osteoarthritis (OA) of the knee and the hip are common conditions in the community, characterized by the degeneration of cartilage and its underlying bone with a high impact on the health-related quality of life [1]. As incidence and prevalence of OA rise with advancing age [2], extended life expectancy will result in greater numbers of OA increasing the financial burden on the health care systems considerably [3, 4].

OA is a complex multifactorial disease [5, 6]. Among the predominant risk factors that contribute to OA are age and weight [6–8]. Several studies examined the association of the body mass index (BMI) as well as other indicators of overweight and obesity (e.g. waist-circumference) with OA of knee and hip [9–12]. While several studies confirmed a significant association between BMI and knee OA but not for hip OA [11, 12], others found associations between BMI and hip OA [9, 10].

Due to the above-mentioned inconsistencies in the literature, it was the aim of the study to further investigate the relationship between obesity (current and historic) and OA of the knee and hip. It was hypothesized that there would be a significant association between the ‘maximum-BMI-over-one’s-lifespan’ (‘maximum-BMI’) and OA of the knee or hip (Hypothesis 1). It was further hypothesized that also the ‘current BMI’ would be linked to OA of the knee or hip (Hypothesis 2). Additionally, as an exploratory approach it was also planned to investigate other weight-related variables [13, 14].

## Materials and methods

This case–control study was approved by the Ethics Committee of the Medical University before commencement of the study. Written informed consent was obtained from all participants before they were enrolled.

We included patients on the waiting list for hip or knee arthroplasty above 50 years of age. Exclusion criteria for the study and the reference group were as follows: (a) patients with OA or endoprosthesis of the hip for patients in the knee group and vice versa, (b) secondary OA due to hip dysplasia, (c) secondary OA due to trauma, (d) secondary OA due to infection, (e) prolonged corticosteroid therapy, (f) previous chemotherapy, (g) impaired cognitive functions, and (h) language barriers.

The reference group consisted of patients: (a) free of hip/knee OA in their medical history and (b) free of hip/knee symptoms. We included patients admitted for other reasons (e.g. upper limb surgery) as well as volunteers recruited via information in general practitioners’ offices, on the hospital’s intranet homepage and in a local newspaper.

All participants underwent a standardized interview to assess their weight at the age of 18 years, their highest and lowest weight in adulthood. It might be speculated whether there would be some recall bias during self-reporting of body weight. However, previous research reported that recall and self-reporting of weight and height are satisfactorily accurate [15, 16].

As objective measurements, participants were weighed on a calibrated personal scale, height was measured with a stadiometer and waist circumferences were determined using a measuring tape. Height and weight were measured wearing light indoor clothing without shoes. The body mass index (BMI) was calculated from height and weight values. The waist circumference was measured over the bare skin on the narrowest circumference between the iliac crest and the costal margin; if no such distinction could be made, the circumference at the height of the umbilicus was taken.

Binary logistic regressions were used to determine predictors for OA group membership and the Enter method to calculate the complete model. All models were adjusted for age and sex. ‘Maximum-BMI’ was used as main indicator and tested for the predictive value. Current BMI was also tested. We evaluated the odds ratios (OR) with their 95% confidence intervals (CI), the *p* values and the Nagelkerke *R* ^2^ (*R* ^2^). Statistical analyses were performed using the SPSS software, version 18.0. The patients’ characteristics were calculated in Excel, Microsoft Office 2003. *p* values (*p*) of < 0.05 were considered significant. In addition to testing our main hypotheses, we used an explorative approach to examine other possible weight-related predictors of OA: we evaluated the effects of weight change (measured as the “maximum-BMI-change” over one’s previous adult life (= “maximum-BMI” −  “minimum-BMI” after the age of 18 years) and as the “adult-BMI-change” (= “maximum-BMI” − “BMI-at-age-18-years”) as well as weight distribution indicators, namely, current “waist-circumference” and “waist-circumference-to-height-ratio” (as they have been linked to a metabolic, leptin-associated pathway of OA [17, 18]).

## Results

99 participants were recruited, 49 women and 50 men. 29 patients were in the knee OA group (18 women and 11 men), 30 in the hip OA group (10 women and 20 men), and 40 in the reference group (21 women and 19 men) (Table 1). While the mean age in the knee OA group was older [mean 71.4 years, standard deviation (SD) 10.2], than in the reference group (mean 65.1 years, SD 7.3)—which was accounted for by adjusting for age in our binary logistic regression models—the mean age in the hip OA group (mean 65.1 years, SD 7.7), was similar to the reference group.

**Table 1:** Patients characteristics

Indicators	Total (*n* = 99)	Hip OA Group (*n* = 30)	Knee OA Group (*n* = 29)	Reference Group (*n* = 40)
Age (years)	66.9 (8.8)	65.1 (7.7)	71.4 (10.2)	65.1 (7.3)
Age BMI min^a^	28.4 (14.9)	29.2 (17.7)	26.3 (11.7)	29.4 (14.9)
Age BMI max^b^	48.3 (17.3)	42.8 (18.6)	52.3 (15.0)	49.4 (17.1)
BMI current^c^	27.3 (4.8)	27.4 (5.5)	29.0 (4.5)	26.1 (4.0)
BMI min^d^	21.7 (2.5)	22.3 (2.9)	22.4 (2.4)	20.7 (1.8)
BMI max^e^	29.5 (4.7)	30.1 (4.7)	31.3 (5.0)	27.7 (4.0)
BMI max change^f^	5.8 (6.6)	4.9 (7.6)	8.1 (5.2)	4.8 (6.3)
BMI max change (1 year)^g^	2.3 (2.5)	1.5 (2.1)	3.3 (3.1)	2.1 (2.1)
BMI change (max—18)^h^	7.0 (4.7)	6.5 (5.3)	8.8 (4.3)	6.0 (4.2)
Waist circumference	96.7 (16.9)	95.7 (22.3)	103.8 (13.4)	92.3 (12.6)
Waist-to-height ratio	58.0 (8.0)	56.0 (12.8)	63.1 (7.3)	54.3 (7.0)

Mean and standard deviation (in brackets) are given

*OA* osteoarthritis

^a^Age BMI min age at BMI minimum

^b^Age BMI max age at BMI maximum

^c^BMI current = weight (kilogram, measured)/height^2^ (meter, measured)

^d^BMI min = “minimum-BMI” = lowest reported weight/height^2^ (according to the questionnaire)

^e^BMI max = “maximum BMI” = highest reported weight/height^2^

^f^BMI max change = “maximum BMI change” = BMI max − BMI min

^g^BMI max change (1 year) = “maximum BMI change within 1 year” = reported max weight in 1 year/height^2^

^h^BMI change (max—18) = “adult-BMI-change” = BMI maximum at age of 18 years (reported)

An increase in ‘maximum-BMI’, increased the odds ratio for both knee OA (OR 1.2; CI 1.1–1.4; *p* = 0.005; *R* ^2^ = 0.36) and hip OA (OR 1.2; CI 1.0–1.3; *p* = 0.027; *R* ^2^ = 0.16) (Hypothesis 1).

The mean current BMI of the knee OA group (mean 29.0, SD 4.5) was higher than in the other groups, which was statistically significant in the regression model when compared to the reference group (OR 1.2; CI 1.1–1.4; *p* = 0.01; *R* ^2^ = 0.31), but not when compared to the hip OA group (OR 1.1; *p* = 0.14). The mean current BMI was slightly higher in the hip OA group (mean 27.4, SD 5.5) compared to the reference group (mean 26.1, SD 4.0), but not statistically significant in the binary logistic regression model (OR 1.0; *p* = 0.312). This means an association of the current BMI to knee OA but not to hip OA (Hypothesis 2).

For our explorative research, we found the following indicators to have a statistically significant predictive power: the only other predictor for hip OA, besides the ‘maximum-BMI’, was a high lowest BMI over the age of 18 years (‘minimum-BMI’) (OR 1.3; CI 1.0–1.7; *p* = 0.022; *R* ^2^ = 0.16). This was also the strongest predictor for knee OA (OR 1.6; CI 1.2–2.2; *p* = 0.003; *R* ^2^ = 0.36). With regards to knee OA, all indicators showed a statistically significant predictive value (Table 2). When comparing the knee OA to the hip OA group directly, the waist distribution indicators showed a significant association with knee OA: the ‘waist-to-height-ratio’ (OR 1.1; CI 1.0–1.2; *p* = 0.032; *R* ^2^ = 0.38) showed a similar predictive value as the ‘waist-circumference’ alone (OR 1.1; CI 1.0–1.1; *p* = 0.038; *R* ^2^ = 0.36).

**Table 2:** Comparison of predictive value if indicators and obesity among hip, knee and reference group

Indicators	HIP OA to reference group				Knee OA to reference group				Knee OA to hip OA group			
	*p* value	OR	95% CI	*R* ^2^	*p* value	OR	95% CI	*R* ^2^	*p* value	OR	95% CI	*R* ^2^
BMI max	0.027	1.154	1.017–1.310	0.16	0.005	1.220	1.063–1.400	0.359	0.241	1.081	0.949–1.231	0.275
BMI max change 1 year	0.329	0.880	0.682–1.137	0.067	0.116	1.187	0.959–1.471	0.234	0.011	1.447	1.086–1.926	0.396
Current BMI	0.312	1.056	0.950–1.172	0.068	0.010	1.201	1.045–1380	0.313	0.138	1.104	0.969–1.258	0.292
BMI change (max—18)	0.392	1.043	0.947–1.150	0.063	0.011	1.186	1.040–1.352	0.317	0.061	1.129	0.994–1.282	0.317
BMI max change	0.178	1.086	0.963–1.224	0.086	0.041	1.165	1.007–1.349	0.272	0.306	1.077	0.935–1.240	0.267
BMI min.	0.022	1.313	1.040–1.657	0.155	0.003	1.583	1.164–2.153	0.36	0.552	1.069	0.857–1.334	0.254
Waist circumference	0.850	1.003	0.972–1.036	0.05	0.002	1.091	1.032–1.153	0.382	0.038	1.056	1.003–1.111	0.364
Waist-to-height ratio^a^	0.682	1.012	0.958–1.069	0.052	0.001	1.184	1.073–1.307	0.437	0.032	1.105	1.009–1.211	0.376

*OR* odds ratio, *95% Cl* 95% confidence interval, *R* ^*2*^ Nagel Kerkes *R* ^2^

^a^Waist-to-height ratio = waist circumference/height

## Discussion

The most important findings of the study were a significant association between OA of both knee and hip with the ‘maximum-BMI’ over one’s lifetime. It is also regarded as important finding that the ‘current BMI’, was associated only with knee OA but not with hip OA. Moreover, the predictive value (OR) of the ‘maximum-BMI’ was greater than that of the ‘current BMI’ in knee OA. The findings are interpreted in a way that the maximum BMI in one’s lifetime is a more appropriate predictor than the current BMI to assess the risk of hip and knee OA.

With regard to the parameter ‘maximum BMI’ over one’s lifetime, we tried to compare our findings with what was previously published. However, to our best knowledge, no previous investigators dealt with the relationship between ‘maximum BMI’ and OA. As the ‘maximum-BMI’ explains only 36% of the variance in our knee OA to reference group model and 16% of the variance in our hip OA to reference group model, further factors need to be investigated and included in a future risk assessment tool like a risk calculator.

‘Current BMI’ was only associated with OA of the knee in our study population. This supports previous research from Reijman et al. and Grotle et al. [11, 12] and contradicts findings from Wang et al. and Lohmander et al. [9, 10]. Obesity was reported as important risk factor for the onset of OA of the knee joint [19–21] and a questionable risk factor for the development of OA of the hip joint [12, 22]. Various studies have demonstrated that the current BMI was related to knee OA and that weight loss improves symptoms and functional capacity [12, 23–25]. Biomechanical studies tried to investigate why the knee joint in overweight persons was more affected by OA than the hip joint. A study by Amiri et al. found, that obesity caused a prolonged activation of quadriceps and gastrocnemii, which can result in a prolonged knee joint contact loading, and thereby may contribute to the predisposition of the knee joint to development and progression of OA [26]. This finding is supported by a study by Pamukoff et al. [27]. They found that obesity may contribute to knee OA because of a greater vertical loading in gait biomechanics [27]. Current BMI was found to be a questionable risk factor for the onset of OA of the hip joint [12, 22]. Overall, in the current literature is consensus, that weight loss is protective against the development of degenerative musculoskeletal conditions [19, 23–25].

In the additional, explorative data analysis, we also found both weight distribution indicators (“waist-circumference” and “waist-to-height-ratio”) to be significant predictors of group membership for the knee OA versus the hip OA group. This data possibly indicates that the knee is more vulnerable to metabolic damage to joint and cartilage, which is associated with a higher intra-abdominal fat mass [28]. Like previous studies [18, 29, 30], we also found an association between these weight distribution indicators with knee OA compared to the reference group. Additionally, we found that the knee seems to be significantly more vulnerable to the amount of intra-abdominal fat than the hip, possibly indicating a stronger leptin-associated pathogenesis of knee OA. ‘Waist-circumference-to-height-ratio’ and ‘waist-circumference’ may help distinguish whether there is a higher risk of getting knee OA than hip OA. Further investigation of these indicators is needed, to confirm and clarify the interpretation of the findings of our exploratory data analyses.

As limitations of our study, a potential selection bias in our reference group has to be acknowledged. It is possible that more health conscious people volunteered in that group. Another limitation is the study design (case–control study) with typical limitation in comparison with prospective studies. Another limitation is that we neglected the effect of leg malalignment in our model. Moreover, current BMI, waist-circumference, and waist-circumference-to-height-ratio measured at study entry could have been biased by limited physical activity associated with OA. It might also be speculated whether there would be some recall bias during self-reporting of body weight. However, previous research reported that recall and self-reporting of weight and height are satisfactorily accurate [15, 16].

Based on our findings, it is concluded that the maximum BMI over one’s lifespan is a better predictor of OA of the hip or the knee than the current BMI. These findings support the use of the most sustainable methods of weight control. The knee joint seems to be more sensitive to obesity as current BMI was associated only with knee OA but not with hip OA.
